# Assessing Eating Disorder Risk: The Pivotal Role of Achievement Anxiety, Depression and Female Gender in Non-Clinical Samples

**DOI:** 10.3390/nu5030811

**Published:** 2013-03-12

**Authors:** Konstantinos C. Fragkos, Christos C. Frangos

**Affiliations:** 1 Centre for Gastroenterology and Nutrition, Division of Medicine, University College London, Rockefeller Building, 21 University Street, London, WC1E 6DE, UK; 2 Department of Business Administration, Technological Educational Institute (TEI) of Athens, Agiou Spyridonos Street, Egaleo 12210, Athens, Greece; E-Mail: cfragos@teiath.gr

**Keywords:** eating disorders risk, female gender, romantic relationships, logistic regression, structural equation modelling

## Abstract

The objective of the present study was to assess factors predicting eating disorder risk in a sample of undergraduate students. A structured questionnaire was employed on a random sample (*n* = 1865) consisting of the following sections: demographics, SCOFF (Sick, Control, One stone, Fat, Food) questionnaire for screening eating disorders and the Achievement Anxiety Test and the Depression, Anxiety and Stress Scale. The students at risk for eating disorders (SCOFF score ≥2) were 39.7%. Eating disorder risk was more frequent in females, students with divorced parents, students who lived alone, students who were seeking a romantic relationship or were married, students who were at a post-secondary vocational institute/college (private-public) educational level and who were more likely to have marks under merit level. Also, the mean scores for the psychological factors of depression, stress and anxiety were higher in students with eating disorder risk. A logistic regression model was produced depicting that depression, stress, female gender, being married and searching for a romantic relationship were risk factors of having an eating disorder risk. The suggested psychological model examined with structural equation modelling signified the role of academic anxiety as an immediate precursor of general anxiety. Hence, college populations in Greece need organized infrastructures of nutrition health services and campaigns to assist in reducing the risk of eating disorders.

## 1. Introduction

The term “eating disorder” refers to a persistent and severe disturbance of eating habits that results in impaired physical health or psychosocial functioning [1]. Anorexia nervosa and bulimia nervosa are the best characterized of the eating disorders [2]. Eating disorders and obesity may coexist, although in clinical practice, most people with an eating disorder have normal or low body weight. Eating disorders are among the potentially lethal psychiatric illnesses and are predominately represented by a mental effect of preoccupation with body weight, shape and diet [3,4]. They frequently occur with other psychiatric disorders, such as depression, substance abuse and anxiety disorders [5]. Although their exact cause is unknown, it is believed that a combination of biological, psychological and/or environmental abnormalities contribute to their development [1,6]. 

Assessing aetiology for eating disorders requires considering multifactorial theories from psychiatry and college health. Initially, the core assumption of eating disorders’ aetiology and maintenance is a dysfunctional system for evaluating self-worth, whereas most people evaluate themselves on the basis of their perceived performance in a variety of domains of life, people with eating disorders judge themselves largely or even exclusively, in terms of their eating habits, shape or weight (and often all three) and their ability to control them [7]. These distinctive and highly characteristic, behavioural and attitudinal features are prominent and well-recognised, as is the dysfunctional system for evaluating self-worth [8]. Jacobi *et al.* [9] classification for the aetiopathology of eating disorders offers a reasonable starting point for assessment of putative risk factors, with the ability to adjust their investigation to certain theories. Additionally, college health scholarship commonly regards the transition to college as a high-risk period for the development of eating disorders, given the notably high rates of dieting, body dissatisfaction and disordered eating among college students, the association between stress and eating disorder symptoms and the typical onset of bulimia nervosa in late adolescence and early adulthood and anorexia nervosa in mid-late adolescence [10,11,12,13]. Prevalence estimates of current eating disorders among college students range from 8% to 17% [1].

In college students’ samples, there is an increasing presence of academic related stress and anxiety. There are now certain studies, along with many case reports or newspaper articles, which report of students (mainly female) who neglect their eating patterns, because of increased academic pressure [14]. Psychological factors have been readily associated with eating disorders, most notably with depression, stress and anxiety [9]. Standard factors that are also present in eating disorders involve female gender, family factors and socio-economic factors [4,6,15]. Female gender has been associated very strongly with the presence of eating disorders [16]. Family dysfunction with a negative intra-family climate has been shown to affect negatively the presence of eating disorders [17,18]. Apart from a negative family status, the status of being in a relationship has been shown to possibly affect the presence of eating disorders [19]. Also, considering that unemployment has been associated many times with other psychiatric disorders, this has not been explored in association with eating disorders [20,21].

These studies point out the significance of addressing eating pathology in college populations, particularly considering the many channels—residential life, academics, extracurricular activities, social networks and health services—by which students can be reached [1]. Understanding populations who are not receiving clinical care is important, as early detection and treatment of eating disorders greatly increases the chances of full recovery. A more detailed picture of how these variables relate to eating disorder risk can help inform efforts to target or tailor intervention strategies on campuses [15,22].

In Greek cohort studies, observations are largely similar. There isn’t an extensive literature in the field yet; however, some conclusions can be drawn [8,23,24,25,26,27,28,29,30,31,32,33,34,35,36]. Studies on adolescents have reproduced international findings where false body image and female gender are positive predictors of eating disorders [24,27,34]. There have been a couple of studies on college student samples, which showed similar results with respect to gender and pointed out the role of anxiety traits [30,32]. However, samples involve specific faculty departments or specific groups. There isn’t a conclusive image of students’ eating disorder risk after adolescents have finished high school in Greece.

Hence, it is important to understand how eating disorder risk varies across student characteristics, such as sex, academic level, family status and interpersonal relationships, as well as employment status. Thus, in this study, we addressed these knowledge gaps using a randomly selected sample of students from post-secondary institutions and public universities in Athens, Greece. First, we estimated the prevalence of eating disorder risk symptoms across subgroups defined by sex, academic level, employment status and family factors, using a standardized instrument. We hypothesized that the prevalence of eating disorder risk symptoms would be higher among women, as in previous studies, whereas we did not have a clear expectation regarding differences by academic level, family or employment status (due to the sparse literature on eating disorder risk associated with these factors in college populations). Second, we estimated the extent to which eating disorder risk symptoms co-occurred with depressive, anxiety and stress symptoms, with particular emphasis on academic stress. We hypothesized that eating disorder risk symptoms would be positively correlated with depression, anxiety, stress and academic related anxiety. Finally, we examined a psychological model under which eating disorder risk could be caused by the presence of these psychological traits.

## 2. Methodology

### 2.1. Participants

The cross-sectional sample survey was conducted between January 2010 and January 2011 among a random sample of students who had graduated from at least high school. This involves post-secondary vocational school students, undergraduate or postgraduate students, drawn randomly from public or private educational institutions in Athens. In Greece, higher educational institutions are of two categories: technological educational institutes (TEI) and higher educational institutes (in Greek, AEI). TEIs were previously polytechnics, now delivering university level education mostly in the technical fields; AEI refers to what is commonly considered as universities. Sampling was based on the techniques suggested by Bartlett *et al.* [37]. Questionnaires were distributed and completed with face-to-face interviews. The sample size chosen was three-times the allowed sample size for representativeness (suggested sample size for 95% confidence level, tolerated margin error 3% and response rate over 80% is 683) [37]. Hence, 1978 questionnaires were distributed to these institutions; 113 were excluded due to incomplete answers. Hence, the final sample size analyzed was 1865. The sample consisted of 45.5% males and 54.5% females, and mean age was 21.2 years. Age distribution is shown in Table 1, along with other demographics regarding family, academic and employment statuses. The present study was approved by the Institutional Board of the organizing institution (TEI of Athens) and was executed following the principles of confidentiality, anonymity and informed consent, as outlined by the Declaration of Helsinki and its subsequent revisions.

**Table 1:** Sample characteristics.

Variable	Frequency	%
*Age*		
16 ≤ age < 18	265	14.2
18 ≤ age < 22	1007	54.0
22 ≤ age < 26	389	20.9
26 ≤ age	110	10.7
NA	4	0.2
*Family status*		
*Do your parents live together?*		
Yes	1469	78.8
No	360	19.3
NA	36	1.9
*Are your parents divorced?*		
Yes	343	18.4
No	1270	68.1
NA	252	13.5
*Whom do you live with?*		
My parents	1114	59.7
Alone, because I work and I am financially independent.	231	12.4
Alone, because I study in a different town from my parents.	407	21.8
Alone, because I study and I want to be independent of control.	108	5.8
NA	5	0.3
*Personal family status*		
Single	1325	71.0
Married	79	4.2
Divorced	24	1.3
Permanent relation, but unmarried	227	12.2
Engaged	48	2.6
Seeking romantic relationship	153	8.2
NA	9	0.5
*Academic status*		
*Current educational institutional level*		
Post-secondary vocational institute/college (private-public)	304	16.3
Higher educational institution-TEI	793	42.5
Higher educational institution-AEI	509	27.3
Postgraduate studies	106	5.7
NA	153	8.2
*Highest educational degree attained till today*		
GCSE/A-levels	1477	79.2
Graduation certificate from post-secondaryvocational institute/college (private-public)	56	3.0
Bachelors	212	11.4
Postgraduate degree	46	2.5
ΝΑ	74	4.0
*If you are a freshman, what was your mark average during your last year of high-school*		
*or during A-levels? (Marks range from 0 = fail to 20 = distinction)*		
mark ≤ 10	56	3.0
10.1 ≤ mark ≤ 14	380	20.4
14.1 ≤ mark ≤ 18	688	36.9
18.1 ≤ mark ≤ 20	114	6.1
ΝΑ	627	33.6
*Mark average during previous term*		
Fail	83	4.5
Pass	364	19.5
Merit	544	29.2
Distinction	170	9.1
ΝΑ	704	37.7
*Employment status*		
*Do you work?*		
Yes	856	45.9
No	954	51.2
NA	55	2.9
*If yes, are you full-time or part-time?*		
Full time	385	20.6
Part time	529	28.4
NA	951	51.0

NA: not available.

### 2.2. Measures

*Demographics*: This section consisted of 12 items with questions on age, gender, family, academic and employment statuses. Results are shown in Table 1.

*SCOFF*: The SCOFF questionnaire is a simple 5-question screening tool for eating disorders. Its acronym is derived from initial wordings in its 5 items [38]. Answers to items are yes or no; an answer of yes to 2 or more of these questions indicates a likely case of anorexia or bulimia [39]. These questions are easy to recall and can guide practitioners in identifying who is at risk for such disorders. Initial testing in the United Kingdom revealed that a threshold of 2 or more positive answers out of 5 gave a 100% sensitivity (95% CI 96.9%–100%) and an 87.5% specificity (95% CI 79.2%–93.4%) [39,40]. It has previously been validated in Greek students [41,42]. It is a reliable and valid screening tool that has been translated into various languages [43,44]. Even though the cut-off point is the same, sensitivity and specificity values are different in these studies. 

*Depression Anxiety Stress Scale*: The Depression Anxiety Stress Scale (DASS) assesses the experience of 42 negative emotional symptoms over the previous week on a 4-point Likert scale, ranging from 0 (does not apply to me) to 3 (applied to me very much) [45]. The DASS was originally developed and validated in Australia, but it has also been validated and translated for use in other countries, such as in the United Kingdom, Malaysia, Arabic countries and Spain among others [46]. The DASS has three sub-scales, namely depression, anxiety and stress subscale, each consisting of 14 items.

Although DASS has been validated in previous Greek studies [47], its psychometric properties are still unclear especially among university students. Thus, it was necessary to perform exploratory factor analysis (EFA) to examine the underlying structure of the scale among our sample of Greek university students. We performed EFA with principal component analysis. Our analysis recognized three factors explaining 46.7% of the total variance. These factors were very similar to the item composition of DASS subscales commonly described, with minor differences in items loading in each subscale and certain items having to be dropped. Results are shown in the Appendix. Because of these differences in items, these factors are represented by the factor scores based on EFA, rather than the sum of items in each subscale. Higher values in each score indicate a higher intensity of the condition. Internal consistency of each factor was very high as well (Depression: Cronbach’s alpha = 0.937, 19 items; Anxiety: Cronbach’s alpha = 0.870, 12 items; Stress: Cronbach’s alpha = 0.861, 9 items).

*Achievement Anxiety Test* (*AAT*): Alpert and Haber [48] designed the AAT to measure facilitating and debilitating test anxiety. The facilitating scale assesses anxiety as a motivator for academic performance and the debilitating scale assesses the degree to which anxiety interferes with academic performance. The whole scale has 19 items. Previous literature indicates satisfactory test-retest reliability, while both facilitating and debilitating anxiety scores were shown to be significant predictors of grade point averages [49]. In the present study, each scale’s Cronbach’s alpha was 0.734 and 0.595 respectively, indicating satisfactory internal consistency.

### 2.3. Statistical Analysis

Demographic information was presented with frequencies and percentages. Univariate analyses were done to examine the factors of the questionnaire associated with risk of eating disorders. Chi-square values or independent sample *t*-tests, degrees of freedom and levels of significance are reported. The effects of depression, achievement anxiety in test and general anxiety, as well as stress were tested as precursors of eating disorder risk using path analysis modelling, wherein the model fit was examined, as well as the significance of the direct and indirect effects. The following indicators were used to assess the goodness of fit of the models: Comparative Fit Index and Root Mean Square Error of Approximation. The chi-square statistic was used for the structural invariance tests to determine significant effect modifiers. The maximum likelihood estimation method for structural equation modelling was used to test the conceptual model, examining the relationships among latent variables [50]. Finally, we performed stepwise logistic regression with the presence of eating disorder risk as the dependent variable and independent variables several demographic and psychological variables. In all calculations, *p*-values under 0.05 were considered significant, unless otherwise stated. All figures and graphs were produced with PASW 18.0 and AMOS 16.0.

## 3. Results

### 3.1. Eating Disorders Risk

The students at risk for eating disorders were 39.7%. Univariate analyses showed that factors associated with the disease at the 5% level were gender, whom they lived with, personal family status, current educational institutional level, depression, anxiety, stress and debilitating test anxiety. Borderline significance (attained at 10% level of significance) was attained with variables the following: whether parents were divorced, average mark during previous term and facilitating test anxiety (Table 2).

**Table 2:** Univariate analyses of eating disorder risk with other variables.

Variables	Eating disorder risk (SCOFF ≥ 2)		Test result
	No	Yes	
**Categorical variables**	***n* (%)**	***n* (%)**	**Chi-square tests**
*Demographic characteristics*			
*Gender*			
Male	561 (50.0%)	288 (38.9%)	*χ*^2^ = 22.134, df = 1, *p* < 0.0001 or = 1.57 (95% CI 1.30–1.90)
Female	562 (50.0%)	453 (61.1%)	
*Age*			
16 ≤ age < 18	143 (12.8%)	122 (16.5%)	*χ*^2^ = 9.185, df = 6, *p* = 0.163
18 ≤ age < 22	631 (56.3%)	376 (50.9%)	
22 ≤ age < 26	230 (20.5%)	158 (21.4%)	
26 ≤ age	117 (10.4%)	8343 (11.2%)	
*Family factors*			
*Do your parents live together?*			
No	204 (18.5%)	156 (21.5%)	*χ*^2^ = 2.376, df = 1, *p* = 0.123, OR = 0.83 (95% CI 0.66–1.05)
Yes	897 (81.5%)	571 (78.5%)	
*Are your parents divorced?*			
No	767 (80.1%)	502 (76.6%)	*χ*^2^ = 2.852, df = 1, *p* = 0.09, OR = 1.23 (95% CI 0.97–1.57)
Yes	190 (19.9%)	153 (23.4%)	
*Whom do you live with?*			
My parents	694 (61.9%)	419 (56.7%)	*χ*^2^ = 11.262, df = 3, *p* = 0.0104
Alone, because I work and I am financially independent	135 (12.0%)	96 (13.0%)	
Alone, because I study in a different town from my parents	242 (21.6%)	165 (22.4%)	
Alone, because I study and I want to be independent of control	50 (4.5%)	58 (7.9%)	
*Personal Family Status*			
Single	828 (74.1%)	496 (67.2%)	*χ*^2^ = 30.728, df = 5, *p* < 0.0001
Married	28 (2.5%)	51 (6.9%)	
Divorced	17 (1.5%)	7 (0.9%)	
Permanent relation, but unmarried	140 (12.5%)	87 (11.8%)	
Engaged	26 (2.4%)	22 (3.0%)	
Seeking romantic relationship	78 (7.0%)	75 (10.2%)	
*Academic factors*			
*Current educational institutional level*			
Post secondary vocational institute/college (private-public)	157 (15.2%)	147 (21.6%)	*χ*^2^ = 14.541, df = 3, *p* = 0.002
Higher education institution-TEI	508 (49.3%)	285 (41.9%)	
Higher education institution-AEI	305 (29.6%)	203 (29.9%)	
Postgraduate studies	61 (5.9%)	45 (6.6%)	
*Highest educational degree attained till today*			
GCSE/A-levels	902 (83.6%)	574 (80.5%)	*χ*^2^ = 3.432, df = 3, *p* = 0.330
Graduation certificate from post secondary vocational institute/college (Private-Public)	32 (3.0%)	24 (3.3%)	
Bachelors	116 (10.8%)	96 (13.5%)	
Postgraduate degree	27 (2.6%)	19 (2.7%)	
*If you are a freshman, what was your average mark during your last year at high-school or during A-levels?*			
mark ≤ 10	35 (4.8%)	21 (4.0%)	*χ*^2^ = 4.388, df = 5, *p* = 0.495
10.1 ≤ mark ≤ 14	217 (30.0%)	163 (31.6%)	
14.1 ≤ mark ≤ 18	412 (57.1%)	276 (53.5%)	
18.1 ≤ mark ≤ 20	58 (8.1%)	56 (10.9%)	
*Average mark during previous term*			
Fail	40 (6.0%)	43 (8.8%)	*χ*^2^ = 6.553, df = 3, *p* = 0.088
Pass	199 (29.8%)	165 (33.5%)	
Merit	330 (49.4%)	213 (43.3%)	
Distinction	99 (14.8%)	71 (14.4%)	
*Employment status*			
*Do you work?*			
No	557 (51.5%)	397 (54.6%)	*χ*^2^ = 1.708, df = 1, *p* = 0.191, OR = 0.88 (95% CI 0.73–1.07)
Yes	525 (48.5%)	330 (45.4%)	
*If yes, are you full time or part time?*			
Full-time	228 (41.6%)	157 (43.0%)	*χ*^2^ = 0.178, df = 1, *p* = 0.673, OR = 0.94 (95% CI 0.72–1.23)
Part-time	320 (58.4%)	208 (57.0%)	
**Continuous variables**	**Mean ± SE**	**Mean ± SE**	***t*-Tests**
Depression	−0.275 ± 0.026	0.404 ± 0.040	*t* = 14.868, df = 1796, *p* < 0.0001, Mean difference (Yes–No) = 0.679
Anxiety	−0.176 ± 0.031	0.265 ± 0.035	*t* = 9.336, df = 1796, *p* < 0.0001, Mean difference (Yes–No) = 0.441
Stress	−0.211 ± 0.028	0.313 ± 0.039	*t* = 11.250, df = 1796, *p* < 0.0001, Mean difference (Yes–No) = 0.524
Debilitating achievement anxiety	32.22 ± 0.20	30.29 ± 0.21	*t* = −6.509, df = 1860, *p* < 0.0001, Mean difference (Yes–No) = −1.93
Facilitating achievement anxiety	27.55 ± 0.15	27.12 ± 0.19	*t* = −1.750, df = 1861, *p* = 0.08, Mean difference (Yes–No) = −0.43

OR: odds ratio; SCOFF: “Sick, Control, One stone, Fat, Food” questionnaire; GCSE: General Certificate of Secondary Education.

Interestingly, eating disorder risk was more frequent in females, students with divorced parents, students who lived alone, students who were seeking a romantic relationship or were married, students who were at post-secondary vocational institute/college (private-public) institutional level and were more likely to have marks under merit level. Also, the means scores for the psychological factors of depression, stress and anxiety were higher in students with eating disorder risk (Table 2).

### 3.2. Stepwise Logistic Regression

A stepwise logistic regression (forward method based on maximum likelihood) [51,52] was conducted to predict the possibility of eating disorders using the factors significantly associated with eating disorder risk from univariate analyses. After four steps, the final model included only four predictors, which were all significant.

A test of the full model against a constant only model was statistically significant, indicating that the predictors as a set reliably distinguished between eating disorder risk and non-eating-disorder risk (*χ*^2^ = 251.284, df = 8, *p* < 0.0001). The model as a whole explained between 13.1% (Cox and Snell *R*^2^) and 17.7% (Nagelkerke *R*^2^) of the variance in eating disorders risk and correctly classified 67.8% of cases. The odds ratios are presented in Table 3. All of the independent variables (in various categories) were significant predictors of eating disorder risk. The model produced depicted that depression, stress, female gender, being married and searching for a romantic relationship were risk factors of having an eating disorder risk. Particularly, female students were 1.60-times more likely than men to be at risk for eating disorders. Also interesting is that married people and people seeking a romantic relationship were 2.53- and 1.64-times more likely to develop a risk for eating disorders. Assessment of interaction terms did not increase the explanatory power of the model, and thus, the main effects are described.

**Table 3:** Stepwise logistic regression results.

	Odds Ratio	Odds ratio 95% CI	*p*-Value
*Depression*	1.82	1.60–2.08	<0.0001
*Stress*	1.18	1.03–1.34	0.014
*Gender*			
Female	1.60	1.30–1.96	<0.0001
Male [Reference group]			
*Personal Family Status*			
Married	2.53	1.51–4.25	<0.0001
Divorced	0.29	0.09–0.92	0.036
Permanent relation, but unmarried	1.03	0.75–1.41	0.874
Engaged	1.44	0.77–2.71	0.253
Seeking romantic relationship	1.64	1.14–2.35	0.008
Single [Reference group]			
Constant	0.45		<0.0001

**Figure 1:**
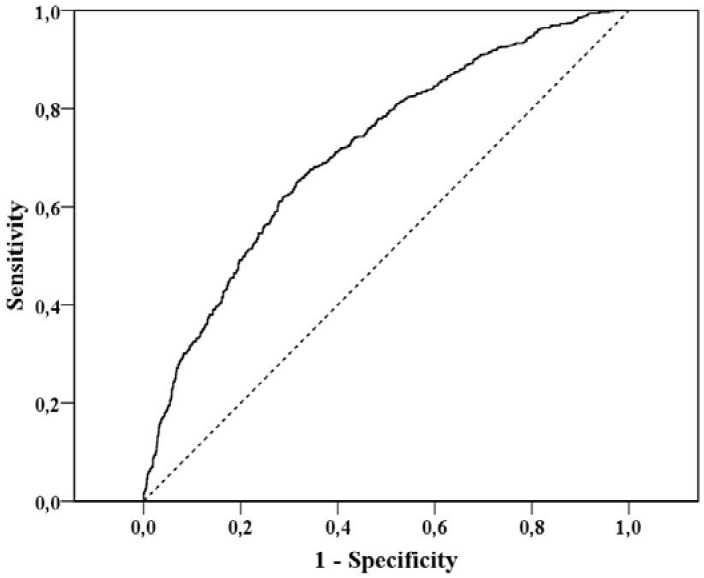
Receiver operating characteristic (ROC) curve examining the discriminatory efficiency of the logistic regression model to detect eating disorder risk.

The logistic regression model was evaluated with the Receiver Operating Characteristic (ROC) curve. In this analysis, the power of the model’s predicted values to discriminate between positive a negative cases is quantified by the Area under the ROC curve (AUC) [53]. The AUC was satisfactory 0.717 (95% CI 0.693–0.741) (Figure 1), signifying a satisfactory discriminatory effect between those with eating disorders and those not.

### 3.3. Suggested Psychological Model

**Figure 2:**
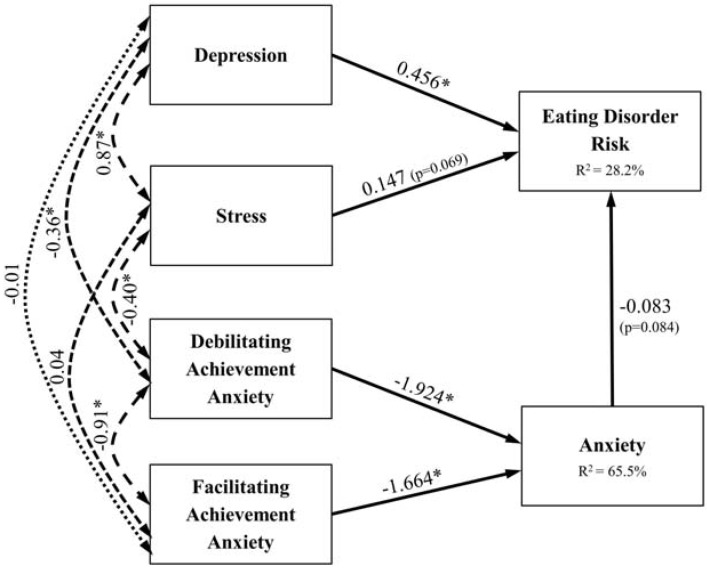
Path model leading to eating disorders risk. Paths from independent to dependent variables depict standardised estimates and double arrows indicate correlations; * *p* < 0.001.

The path model presented adequate fit (Root Mean Square Error of Approximation =0.041, Comparative Fit Index =0.847, *χ*^2^ = 8166.048, df = 1941, *p* < 0.0001). The paths from debilitating and facilitating anxiety leading to anxiety were significant and explained 65.5% in the variance of anxiety (Figure 2). The standardized total effects of these two types of test anxiety were positive predictors of eating disorders, albeit only weakly (0.138 and 0.160) (Table 4). The paths leading to eating disorders risk were all significant at the 10% level of significance, but only depression showed a highly significant effect (path coefficient =0.456, *p* < 0.001). This model seems to explain 28.25% in the variation of eating disorders. Stress was also a moderately positive predictor of eating disorders, but anxiety results are not so conclusive, due to low standardized estimate and non-significant value at the 5% level of significance.

**Table 4:** Path modelling results.

Dependent variable	Pathprecursor	Unstandardised estimate	Standardised estimate	*p*-Value	*R*^2^
*Eating Disorder Risk (SCOFF ≥ 2)*	Depression	0.212	0.456	<0.001	0.282
	Stress	0.072	0.147	0.069	
	Anxiety	−0.046	−0.083	0.084	
	Debilitating Achievement Anxiety	0.087	0.160		
	Facilitating Achievement Anxiety	0.131	0.138		
*Anxiety*	Debilitating Achievement Anxiety	−1.877	−1.924	<0.001	0.655
	Facilitating Achievement Anxiety	−2.828	−1.664	<0.001	

## 4. Discussion

In the present study, we performed an extensive sample survey of students who have finished high-school and are attending higher education or post-secondary vocational institutes. Findings are interesting, since they for the first time pointed out the role of employment status with eating disorder risk. This was not maintained in the multivariate models. Family status was also a strong predictor of eating disorder risk, with being married or being in a relationship being strong predictors. Academic sources of anxiety were not retained in the stepwise logistic regression, but played a role in the suggested psychological pathway.

So, how are these results interpreted? The suggested pathway examined with structural equation modelling gives a satisfactory explanation. Academic anxiety explained 65.5% of anxiety in general. Anxiety in general was higher in subjects with eating disorder risk, but wasn’t a significant predictor in the logistic regression model; this, however, should not be considered as negative, since anxiety was highly correlated with stress and depression, which were strong positive predictors of eating disorder risk in both the pathway and the logistic regression model.

Other results showed that being married was considered a strong predictor of risk of eating disorders. We haven’t explored relationship quality or marital stress *per se* to provide an explanation for this finding. However, other studies have done so [19,54]. Kiriike *et al.* [55] found that 69% of the Japanese female patients with an eating disorder in their study developed the illness due to marital problems, separation or divorce. These results indicate, as might be expected, that it is marital problems that lie at the heart of the association between marriage and disordered eating. From the present data, the odds ratio for the interaction of stress with being married was 2.98 (95% CI 0.97–9.18, *p* = 0.057), which was borderline significant; however, it indicates that both variables together increase the risk of eating disorders [56].

The contribution of the present study to eating disorders scholarship is three-fold. It initially gives an image of college health in Greece, where the risk of eating disorders is prevalent in around 40% of the student population and replicates established findings that eating disorders are associated with female gender and depression/stress. Next, it connects academic activities with the risk of eating disorders, by assessing the impact of achievement anxiety on eating disorder risk. Although academic anxiety was an important component of anxiety in general, it did not affect directly eating disorder risk. Thirdly, married couples and people seeking a romantic relationship were more probable to have a risk of eating disorders; this is possibly explained due to marital or relationship stress. 

Nevertheless, the present study has certain limitations. The major limitation is that no diagnostic data was available against which to validate the eating disorders screening. Secondly, the cross-sectional nature of the study can establish arguments against causality between variables, for which a longitudinal approach would be more appropriate to address. Finally, the data was collected only from college students, who represent a portion of the general population. Thus, a study of selecting a larger general population sample will increase generalisability and also the validity of the study. 

So, eating disorder risk is prevalent among student populations in Greece. At the moment infrastructures for dealing with these are not present in Greek colleges. Although psychological services are present, they are treated with mistrust, because there is the fear of stigmatization of having a mental disorder [33,57,58]. It might prove necessary in the future to screen college freshman with the SCOFF questionnaire upon entry to university. The professional services include health clinics in psychiatric hospitals and certain eating disorder clinics in paediatric hospitals in Greece. These specialised centres need to involve primary care centres (in Greece mainly general physicians—*pathologoi*) for assessing metabolism parameters that could indicate disordered eating (e.g., albumin, protein, thyroid function tests, plasma cortisol, *etc.*). Finally, taking into account the effects of eating disorders on physical and emotional health, it is suggested that prevention programs are in need. The effect of the mass media on the advent of eating disorders has been discussed extensively [35]. However, campaigns showing the clinical importance of eating disorders, as well as the long term effects on people, should be outlined and be considered necessary. These campaigns will assist in alleviating stigmatization associated with these disorders. Once these infrastructures are in place, the road towards professional therapy will seem more natural and less agonizing.

## 5. Conclusions

The present study is one of the largest in the literature assessing eating disorders risk in non-clinical samples. It presented evidence suggesting that female gender, interpersonal relationships and achievement anxiety have a significant association with eating disorder risk; certain of these associations have been observed for the first time in international literature. These results suggest a need for monitoring eating disorder risk in non-clinical populations by attentively identifying risk factors and for the Greek society specifically, the need for more active prevention measures.

## Appendix: Exploratory Factor Analysis of DASS

The 42 items of DASS were subjected to principal components analysis (PCA) using PASW 18.0. Prior to performing PCA, the suitability of data for factor analysis was assessed. Inspection of the correlation matrix revealed the presence of many coefficients 0.300 and above. The Kaiser-Meyer-Olkin value was 0.976, exceeding the recommended value of 0.600 [59,60], and Bartlett’s Test of Sphericity reached statistical significance [61], supporting the factorability of the correlation matrix.

PCA revealed the presence of four components, with eigenvalues exceeding 1, explaining 37.3%, 6.1%, 3.2% and 3.0% of the variance respectively. An inspection of the scree plot revealed a clear break after the third component. Using Cattell’s [62] scree test, it was decided to retain three components for further investigation. This was further supported by the results of parallel analysis, which showed only three components, with eigenvalues exceeding the corresponding criterion values for a randomly generated data matrix of the same size (42 variables *×* 1865 respondents).

The three component solution explained a total of 46.7% of the variance. To aid in the interpretation of these, three components Oblimin Rotation was performed. This method was preferred, because of the high positive correlations between components (Depression and Anxiety: 0.472; Depression and Stress: 0.622; Anxiety and Stress: 0.506). This component solution is in accordance with the original factor analyses of DASS, producing three components depicting depression, anxiety and stress, but with slight differing in certain items and having to drop two items (Table A1). Thus, factor scores were chosen to depict these latent factors instead of item sums for each component.

**Table A1:** Oblimin rotation results of three factor solution of DASS items.

Factor	Item		Pattern Coefficients			Structure Coefficients			Communalities
			Components			Components			
			1	2	3	1	2	3	
Depression	38	I felt that life was meaningless	**0.802**	−0.145	0.078	**0.782**	0.273	0.503	0.627
	17	I felt I wasn’t worth much as a person	**0.792**	0.00002	−0.024	**0.778**	0.362	0.469	0.605
	34	I felt I was pretty worthless	**0.754**	−0.060	0.087	**0.78**	0.340	0.525	0.613
	21	I felt that life wasn’t worthwhile	**0.745**	−0.102	0.108	**0.764**	0.304	0.519	0.594
	37	I could see nothing in the future to be hopeful about	**0.743**	−0.015	0.002	**0.738**	0.337	0.457	0.544
	10	I felt that I had nothing to look forward to.	**0.726**	0.018	−0.018	**0.723**	0.351	0.442	0.523
	16	I felt that I had lost interest in just about everything	**0.690**	0.030	0.029	**0.722**	0.370	0.473	0.523
	26	I felt downhearted and blue	**0.655**	−0.033	0.135	**0.723**	0.344	0.526	0.533
	31	I was unable to become enthusiastic about anything	**0.637**	0.168	−0.049	**0.686**	0.443	0.432	0.49
	30	I feared that I would be “thrown” by some trivial, but unfamiliar task	**0.546**	0.138	0.074	**0.657**	0.433	0.484	0.455
	24	I couldn’t seem to get any enjoyment out of the things I did	**0.504**	0.212	0.098	**0.665**	0.499	0.519	0.491
	42	I found it difficult to work up the initiative to do things	**0.467**	0.114	0.149	**0.614**	0.410	0.498	0.408
	11	I found myself getting upset rather easily	**0.461**	0.389	−0.106	**0.578**	0.553	0.378	0.442
	5	I just couldn’t seem to get going	**0.423**	0.057	0.256	**0.609**	0.386	0.548	0.420
	36	I felt terrified	**0.421**	0.100	0.262	**0.631**	0.431	0.574	0.459
	18	I felt that I was rather touchy	**0.398**	0.294	0.024	**0.552**	0.494	0.420	0.375
	40	I was worried about situations in, which I might panic and make a fool of myself	**0.373**	0.220	0.187	**0.593**	0.491	0.531	0.429
	35	I was intolerant of anything that kept me from getting on with what I was doing	**0.316**	0.238	0.157	**0.527**	0.467	0.475	0.353
	39	I found myself getting agitated	**0.310**	0.229	0.299	**0.604**	0.526	0.607	0.489
Anxiety	29	I found it hard to calm down after something upset me	0.012	**0.721**	−0.013	0.344	**0.720**	0.359	0.519
	22	I found it hard to wind down	0.051	**0.680**	−0.010	0.365	**0.699**	0.365	0.490
	33	I was in a state of nervous tension	−0.016	**0.636**	0.153	0.379	**0.706**	0.465	0.514
	8	I found it difficult to relax	−0.124	**0.635**	0.198	0.299	**0.677**	0.443	0.480
	6	I tended to overreact to situations	0.084	**0.630**	−0.047	0.352	**0.646**	0.324	0.421
	12	I felt that I was using a lot of nervous energy	0.034	**0.627**	0.028	0.347	**0.657**	0.366	0.434
	14	I found myself getting impatient when I was delayed in any way (e.g., elevators, traffic lights, being kept waiting)	−0.154	**0.598**	0.129	0.208	**0.591**	0.335	0.364
	9	I found myself in situations that made me so anxious, I was most relieved when they ended	−0.062	**0.578**	0.070	0.254	**0.584**	0.324	0.344
	1	I found myself getting upset by quite trivial things	0.027	**0.563**	−0.036	0.270	**0.557**	0.266	0.311
	27	I found that I was very irritable	0.234	**0.474**	−0.013	0.450	**0.578**	0.372	0.375
	13	I felt sad and depressed	0.307	**0.460**	0.018	0.535	**0.614**	0.442	0.455
	32	I found it difficult to tolerate interruptions to what I was doing	0.257	**0.438**	−0.016	0.453	**0.551**	0.365	0.352
Stress	41	I experienced trembling (e.g., in the hands)	0.041	0.003	**0.739**	0.502	0.396	**0.766**	0.588
	7	I had a feeling of shakiness (e.g. legs going to give away)	−0.001	0.018	**0.711**	0.450	0.378	**0.719**	0.518
	25	I was aware of the action of my heart in the absence of physical exertion (e.g., sense of heart rate increase, heart missing a beat)	0.028	0.081	**0.668**	0.482	0.433	**0.727**	0.534
	4	I experienced breathing difficulty (e.g., excessively rapid breathing, breathlessness in the absence of physical exertion)	0.037	0.041	**0.660**	0.467	0.392	**0.704**	0.498
	19	I perspired noticeably (e.g., hands sweaty) in the absence of high temperatures or physical exertion	−0.101	0.117	**0.586**	0.319	0.366	**0.583**	0.352
	2	I was aware of dryness of my mouth	0.097	0.052	**0.577**	0.481	0.390	**0.664**	0.450
	23	I had difficulty in swallowing	0.303	−0.126	**0.528**	0.572	0.285	**0.653**	0.483
	15	I had feeling of faintness	0.336	−0.133	**0.490**	0.578	0.274	**0.632**	0.468
	20	I felt scared without any good reason	0.208	0.199	**0.393**	0.546	0.496	**0.624**	0.458
(dropped)	3	I couldn’t seem to experience any positive feeling at all	0.297	0.150	0.297	0.553	0.440	0.558	0.396
(dropped)	28	I felt I was close to panic	0.261	0.230	0.294	0.553	0.502	0.573	0.428
